# An exploratory analysis of the regionalization policy for the recruitment of health workers in Burkina Faso

**DOI:** 10.1186/1478-4491-12-S1-S6

**Published:** 2014-05-12

**Authors:** Seni Kouanda, W Maurice E Yaméogo, Valéry Ridde, Issa Sombié, Banza Baya, Abel Bicaba, Adama Traoré, Blaise Sondo

**Affiliations:** 1 Institut de recherche en sciences de la santé (IRSS), Ouagadougou, Burkina Faso; 2 Institut africain de santé publique (IASP), Ouagadougou, Burkina Faso; 3 Centre de recherche du Centre hospitalier de l’Université de Montréal, Montréal, Canada; 4 L'Institut supérieur des sciences de la population (ISSP), Ouagadougou, Burkina Faso; 5 Institut national de la statistique et de la démographie (INSD), Ouagadougou, Burkina Faso; 6 Société d'études et de recherche en santé publique (SERSAP), Ouagadougou, Burkina Faso; 7 UFR/SDS, Université de Ouagadougou, Ouagadougou, Burkina Faso

**Keywords:** health workers, public policy, evaluation, implementation, travailleurs de la santé, politique publique, évaluation, mise en oeuvre

## Abstract

**Background:**

Health personnel retention in remote areas is a key health systems issue wordwide. To deal with this issue, since 2002 the government of Burkina Faso has implemented a staff retention policy, the regionalized health personnel recruitment policy, aimed at front-line workers such as nurses, midwives, and birth attendants. This study aimed to describe the policy’s development, formulation, and implementation process for the regionalization of health worker recruitment in Burkina Faso.

**Methods:**

We conducted a qualitative study. The unit of analysis is a single case study with several levels of analysis. This study was conducted in three remote areas in Burkina Faso for the implementation portion, and at the central level for the development portion. Indepth interviews were conducted with Ministry of Health officials in charge of human resources, regional directors, regional human resource managers, district chief medical officers, and health workers at primary health centres. In total, 46 indepth interviews were conducted (February 3 - March 16, 2011).

**Results:**

Development

The idea for this policy emerged after finding a highly uneven distribution of health personnel across urban and rural areas, the availability of a large number of health officers in the labour market, and the opportunity given to the Ministry of Health by the government to recruit personnel through a specific budget allocation.

Formulation

The formulation consisted of a call for job applications from the Ministry of Health, which indicates the number of available posts by region.

The respondents interviewed unanimously acknowledged the lack of documents governing the status of this new personnel category.

Implementation

During the initial years of implementation (2002-2003), this policy was limited to recruiting health workers for the regions with no possibility of transfer. The possibility of job-for-job exchange was then approved for a certain time, then cancelled. Starting in 2005, a departure condition was added. Now, regionalized health workers can leave the regions after undergoing a competitive selection process.

**Conclusion:**

The policy was characterized by the absence of written directives and by targeting only one category of personnel. Moreover, there was no associated incentive—financial or otherwise—which poses the question of long-term viability.

## Abstract (fr)

**Contexte:**

Le maintien en poste du personnel de la santé dans des régions éloignées est un des principaux problèmes des systèmes de santé partout dans le monde. Pour tenter de régler ce problème, le gouvernement du Burkina Faso a mis en oeuvre depuis 2002 une politique de rétention du personnel dite politique de recrutement régionalisé du personnel de la santé qui concerne les agents de première ligne que sont le personnel infirmier, les sages-femmes et les accoucheuses. Le présent article a pour objectif de décrire le processus d’émergence, de formulation et de mise en oeuvre de cette politique de régionalisation du recrutement du personnel de la santé au Burkina Faso.

**Méthodes:**

Nous avons mené une étude qualitative. L’unité d’analyse est une étude de cas unique avec plusieurs niveaux d’analyse. L’étude a été menée dans trois régions éloignées du Burkina Faso pour la mise en oeuvre et de façon centralisée pour l’émergence. Des entrevues approfondies ont été menées avec des fonctionnaires du ministère de la Santé qui étaient ou ont été responsables des ressources humaines, des directeurs régionaux, des gestionnaires régionaux des ressources humaines, des médecins-chefs de districts et des travailleurs de la santé dans des centres de soins de santé primaires. Au total, 46 entrevues approfondies ont été menées avec des intervenants de différents groupes entre le 3 février et le 16 mars 2011.

L’idée de cette politique a émergé à la suite du constat de la répartition très inégale entre milieux urbains et ruraux du personnel de la santé d’une part, et d’autre part de la disponibilité d’un grand nombre d’agents de santé sur le marché du travail et de la possibilité offerte au ministère de la Santé de recruter du personnel grâce à l’allocation d’un budget spécifique par le gouvernement.

La formulation consistait en un appel de candidature du ministère de la Santé qui indiquait le nombre de postes disponibles par région.

Les participants rencontrés sont unanimes pour reconnaitre l’inexistence de lignes directrices concernant le statut de cette nouvelle catégorie de personnel.

Au cours des premières années de sa mise en oeuvre (2002-2003), cette politique se résumait aux recrutements du personnel pour les régions sans aucune possibilité de départ. La possibilité d’effectuer une permutation poste pour poste a été acceptée un certain temps, puis a de nouveau été supprimée. À partir de 2005, une condition de départ a été ajoutée. Désormais, les agents régionalisés peuvent quitter les régions après l’admission à un concours professionnel.

**Conclusions:**

Cette politique est limitée par l’absence de lignes directrices écrites et par le fait qu’elle ne s’adresse qu’à une catégorie de personnel. De plus, aucun incitatif, financier ou non, n’y a été associé, ce qui pose le problème de sa viabilité à long terme.

## Background

Human resources for health are essential to national health systems' achievement of the Millennium Development Goals [1-3]. In low-income countries, the current health human resources situation is characterized by a shortage of qualified workers and by an unequal distribution of existing personnel. In many of these countries, there is a visible imbalance in human resources between the public and private sectors, and between reputedly disadvantaged rural and urban areas [2-4].

Health personnel recruitment and retention policies in rural and remote areas are well known. According to several authors [5-8], there are some major strategies to retain personnel in remote underserved areas. The first strategy is related to recruitment of workers of rural origin and trained in schools in their communities [3,9,10]. The strategy aims to recruit and train health personnel in a rural setting using training programs adapted to a rural setting. This strategy was helpful in Thailand where the distribution of its health human resources was considerably improved. In Africa, countries such as Ghana, Ethiopia, and Kenya have introduced community approaches in the new schools of medicine, but short or medium-term evaluations have not yet been conducted [6,9,10].

The second strategy is based on financial incentives [5,6,10-14]. The incentives vary by country, involving substantial compensatory wages for the renovation of housing, or paying for tuition fees for the health workers’ children, like in Zambia, or compensatory wages in rural areas like in South Africa, Nigeria, and Niger [15,16]. This strategy has been shown to have positive effects on the distribution of health workers in rural areas [6,10].

Regulatory measures, including compulsory service, constitute the third strategy for retaining health personnel. Frehywot *et al.* classified these measures into three categories: condition of service/state employment programme (service in rural areas for a set number of years if need to be recruited); compulsory service with incentives (bonus, housing, education opportunities, licence to practice); and compulsory service without incentives [17]. More than 70 countries around the world have compulsory service, but the results show that in most cases, health officers leave the rural area as soon as they have completed their compulsory service period [6,17].

The fourth strategy involves personal and professional support, which includes improving the working and living conditions of health personnel, as well as career development programs [18]. The Zambian Health Worker Retention Scheme included housing renovations and payment of children’s tuition fees in its package [11,12]. There is little available evidence on strategies to improve working conditions and job satisfaction [6,10].

Most of these strategies were documented in Latin America, Australia, the United States, and Southern Africa, and by concerned primarily physicians [5,6,9,11,12,14,17,19,20]. Although there are several health personnel recruitment and retention strategies in West Africa, these strategies have rarely been evaluated. There are limited data from Ghana, Senegal, and Mali [13,16,21].

The aim of this study is to describe the process of development, formulation, and implementation of the regionalization policy for the recruitment of health personnel in Burkina Faso.

### Context

Burkina Faso, a French-speaking country in West Africa with limited resources, has implemented a policy for the regionalization of health personnel recruitment. The aim of the policy was to reduce the unequal distribution between urban and rural areas. Like other developing nations, Burkina Faso is grappling with a shortage of health care human resources and uneven distribution of those resources between urban and rural areas. The country’s two largest cities (Ouagadougou and Bobo-Dioulasso) accounted for 53.7% of the country’s physicians, 57.3% of the midwives, 59% of the pharmacists, and 33% of the nurses, all categories combined, even though these cities account for only 10% of the country’s population [22]. The aim of the policy was to recruit health workers specifically for rural areas, with completion of a competitive selection process as the only way to leave the region of recruitment.

## Methods

### Type of study

This is an exploratory study of a public policy. A public policy is defined as a process to regulate situations that present problems in the distribution of resources [23]. In this case, it is the distribution of health human resources. These public policies are comprised of three sub-processes: development, formulation and implementation [24], which produced different anticipated effects. Furthermore, these public policies are not linear; they interact and quite often adapt to events and contexts [25], since social actors play a key role [26].

This research covered a description of the process of the development, formulation, and implementation of this policy. This helped us to better understand all aspects of introducing regionalization to Burkina Faso.

We conducted a single case study with several levels of analysis [27]. The regionalized health personnel recruitment policy is the case studied. The different levels of analysis are the national and central level for analysis of the development and formulation, and the regional and district level, for analysis of implementation.

### Site selection

**Figure 1:**
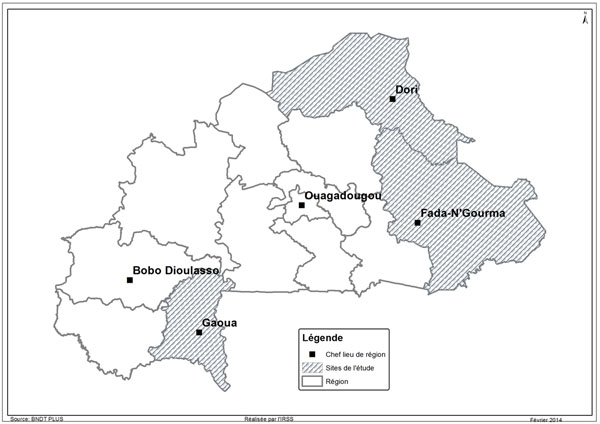
Map of study area

Burkina Faso has 13 health regions. We conducted this study on three defined remote areas, i.e., those with one or more of the following characteristics: remoteness from the country’s two major cities (Ouagadougou and Bobo-Dioulasso), hard to reach, and harsh climate. As such, the Sahel, East, and Southwest regions were selected to analyse the implementation of the policy (see Figure 1).

Two districts from each of the three health regions were included in the study: the district in the region’s administrative centre (urban setting) and one rural district randomly selected among the districts in the region.

### Data collection

Public policy can be analyzed by examining not only the process but also the people who take part in it [27,28]. Four strategic groups of actors [29] are thus concerned, based on their expertise (specialized or non-specialized) and their position related to the government (internal/external) [24]. The research took this aspect of the policy into account by incorporating the viewpoints of several actors, including managers at various levels, the officers concerned by the policy at the primary health care centres (called CSPS), and civil society actors.

For each group, participants were purposively sampled. At the central level, individual interviews were held with five managers (former secretary generals, human resource officer, ministry advisor, director general of health) who were or are in charge of human resource matters for the Ministry of Health. At the regional and district levels, the district chief medical officers and supervisors of human resource managers were interviewed.

Health workers were interviewed in the first line health centres. In each district, the primary health care centre farthest from the district hospital and/or most difficult to reach was selected, and all health workers present at the health centre the day of the survey were interviewed.

Data was collected from February 3 to March 16, 2011. An interview guide covering various topics was developed for each target group. The topics covered varied depending on the type of source, and primarily cover policy content: context of the development and policy formulation, implementation mechanism and difficulties, perception of policy (positive and negative).

All the interviews were recorded with the consent of the interviewees.

### Data analysis methods

The data was analyzed in accordance with our conceptual framework—a framework analysis of the three policy sub-processes [30]. The analysis was conducted using Nvivo 9 software**.**

## Results

### Sample of the qualitative investigation

Indepth interviews were conducted with 46 key informants from various groups (Table 1). In the group of decision makers, seven interviews were held with actors at the central level.

Six interviews were held with the regional group of managers. Seven interviews were held with the district managers. Twenty-four interviews were held with the group of health officers affected by the policy. Two union representatives were also interviewed.

**Table 1:** Characteristics of interviewees

Groups	Male	Female	Total
Decision makers Regional managers District Managers Health union representative Health workers Nurse’s aid Nurse Midwife Midwife Assistant	7 4 6 2 14 4 10 0 0	0 2 1 0 10 0 4 1 5	7 6 7 2 24 4 14 1 5

Total	47	23	46

### Emergence of the regionalization of recruitment policy

Although the distribution of health personnel among the different regions in the country has always been a concern for Ministry of Health officials, the issue emerged as a public problem in 2000 after the establishment of the Millennium Development Goals (MDGs), notably in relation to Goal 5 on the reduction of maternal mortality. It became evident that this goal could not be attained without qualified personnel in remote areas, as one of the lead officials at the time confided:

“*Practically all the midwives were in the city; yet we need midwives outside of the city to reduce maternal mortality if we hope to achieve MDG 5. From that moment on*, *we decided to recruit midwives for the regions”* (decision maker)

The idea to regionalize the recruitment of health personnel emerged in the Ministry of Health at the Secretary General office based on the identification of this problem and of several windows of opportunity.

The first window of opportunity was the availability of a large number of already-trained officers who wanted to join the public service. In fact, access to health care vocational schools (e.g., for nurses, birth attendants, and midwives) is conditional upon completing the competitive selection process organized annually by the Civil Service Ministry. Following training, the new health officers automatically enter the public service and are assigned to the different regions by the Ministry of Health. However, since the early 2000s, health care training schools, formerly open only to personnel who completed the State’s competitive selection process or the professional exam, were opened to qualified registrants, i.e., applicants who chose not to compete or did not compete successfully but who are committed to paying for their training. Unlike those who passed the entrance exam, qualified registrants did not join the public service upon completing their studies.

In doing so, some were able to receive training for various medical professions, including nurse, midwife, and birth attendant. This system was implemented without a policy to absorb the new graduates such that, at one point, the Ministry of Health found itself overwhelmed by applications to join the public service.

The second window of opportunity was the opportunity given to the Ministry of Health, by the government, to recruit personnel based on needs by allocating a specific budget through what are referred to as ‘the new measures’. Since 2000, the government has allocated additional resources to these two ministries to recruit personnel apart from the recruitment organized by the Civil Service Ministry. Hence, starting in 2002, the Ministry of Health decided to recruit health workers (nurses, midwifes) using these budget allocations to address the shortfall issue limiting the operation of a certain number of districts. For one official:

“*The idea of regionalizing the recruitment of workers came out of the finding that some regions were devoid of personnel. We knew that there are people who enrolled individually. They paid*, *and now they are coming to submit applications to the Ministry; while waiting to truly revise the assignment system and to be able to get more health workers*, *we will ask these people whether they want to go work in the regions. It was from that moment that recruiting was done to benefit the regions. We started to recruit already-trained personnel*.” (decision maker)

### Formulation of the regionalization of recruitment policy

The option of regionalized recruitment came about after several meetings in 2001. However, the process remained limited only to central Ministry of Health officials. Neither the regional and district officials, nor the organizations representing health personnel were involved in the formulation process.

The Ministry of Health conducts the regionalized recruitement by a statement which clearly states by region, the number of positions available. This statement specifies that the regionalized staff is recruited to serve only in the area chosen by the candidates. But there is no policy document that specifies the content of this policy, recruitment conditions, departure arrangements in the region, and the career plan for this staff.

The key informants interviewed unanimously acknowledged the absence of written directives governing the status of this new category of worker. One former official explains the reasons for this undefined status:

*“We didn’t for a very simple reason: for us this was not something that was going to last; it was temporary. It would last only the time that it took the State to train the maximum number of health officers. What was important was creating a de facto situation with the public service*, *and the funds to get personnel in underserved regions. Well*, *now*, *was it necessary to get it in writing and make a sort of administrative innovation out of it? No*, *we didn’t think it was worth it.*”

This lack of formalized policy content would have repercussions at the implementation stage.

### Implementation of the regionalization of recruitment policy

Regionalized officers are recruited through a competitive process. This process is organized by the Civil Service Ministry, through a call for applications, for positions in the different regions. These positions were previously determined by the Ministry of Health, which plans this recruitment while developing the annual budget and provides the list of positions to be filled by region.

The recruitment was organized centrally and the first process took place in 2002. Since then, more than ten competitive processes have been organized and, according to data from the Ministry of Health’s Studies Branch, 3 567 health officers have been recruited for underserved regions.

Regionalization went through several phases of implementation. In the initial years (2002-2004), the policy was limited to the recruitment of workers for the regions, without any possibility of moving to another region. Then, the possibility of a job-for-job exchange of posts was approved for a certain time, and then cancelled. Starting in 2005, a transfer condition was added. Now, regionalized officers can leave the regions after going through a competitive selection process.

Implementation is limited by the absence of clear written guidelines on the administrative management of these workers. The consequence was a chaotic implementation in the very beginning (2002-2003). One manager describes the piecemeal implementation as follows:

“*It must be said that this also validates the idea that it was not properly prepared. There was a lot of haste*, *and the first ones to be recruited and sent to the field*, *I think that at that time the HR department did not even know how to manage them*, *and the problems were handled from day to day. The HR would examine a given situation as it would come up*, *see with the Public Service what was appropriate to do; so if a problem was handled in a way*, *from then on if this problem reoccured*, *it would be handled that way*, *…”* (Regional manager)

Another situation that reveals the implementation difficulties related to the lack of written guidelines was the differential treatment, by region, of the possibility for officers with the same profiles to move to another region through the exchange of posts. This was acknowledged by one human resources manager:

“*For example*, *there are situations that arise and one does not know how to manage them. One of these situations is the exchange. They come and say*, *“I am going to swap with someone.” They are told that they cannot swap*, *but in reality there is no written rule that says that they cannot exchange posts*”.

Variations in the implementation of the exchange of posts system are observed. In fact, while in the Sahel and Southwest regions exchange was not possible, officers have been able to leave their region of recruitment through an exchange.

In addition to the absence of a policy document, there was a lack of information between the central level and the regional and district levels involved in implementation. Initially, regional officers were sent to the regions, yet managers did not have sufficient information on their status. This situation allowed some officers to leave the regions, unbeknown to their managers.

Almost ten years after implementation of this policy (2002-2012), this shortcoming has not yet been corrected, as confirmed by this district manager:

“*All I know is that for some years*, *we have been sent officers and they say that they cannot leave the region... I tried to get information but could not find a single official document that explains this process*”.

All managers at all levels (central, regional, and district), acknowledged that they are under pressure from administrative authorities and elected officials, who intervene so that their relatives are not assigned too far from the urban centres.

Regionalized health officers criticized a form of “injustice” that makes them second-class employees. This injustice was felt even more due to the fact that regionalized personnel, unlike those recruited through the direct competitive process, had paid out of their own pocket for their training. A regionalized officer said:

“*I believe that everyone must be treated the same way. I do not understand why qualified registrants*, *who paid for their education out of their own pockets*, *do not have the same rights as the direct ones who are free in all their movements”*.

This opinion was also shared by certain managers, as one conceded:

“*The main problem is that in the long run*, *if we insist that they cannot be assigned anywhere else except over there*, *it becomes an injustice because these officers had the same training; they are doing the same job. The administration must find a strategy so that both [the regionalized and non-regiolalized officers] can have the same rights*, *because there is discrimination*”.

However, for others, the officers’ position has no merit in the sense that they all knew the implications of taking part in the competitive process. The health officers consider that though they knew, the need for employment was so great that they did not weigh the long-term constraints. One officer declared:

*“Since we needed work*, *we chose*, *but once we arrived*, *honestly*, *we realized that it was not easy”.*

As the labor unions representatives, the health officiers consider that with this policy, the Ministry is forging ahead when it comes to human resources management. According to one labor union representative, the policy was put in place because the Ministry of Health was unable to apply the written directives on assignments. For this labor union representative, if every officer spent six years in a rural location, as set out in the written directives, there would have been no need for this policy.

## Discussion

During this study, the analysis was limited by a major difficulty owing to the absence of policy documents, requiring a reconstruction to establish the policy content, with all the attendant imprecision of such a process.

The regionalization of health personnel recruitment that has been in effect in Burkina Faso over the last ten years was not defined by legislation and regulations. The initiators indicated that it was not necessary to formalize this decision in a document, as it was understood that this measure was intended to be temporary. It is true, as indicated by some actors [24], that public policies may exist without a law or regulation being produced. A simple call for job application from a minister could be used as a reference in the development of a procedure. Although, in the eyes of these initiators, the regionalization of health personnel recruitment was not a policy, it was analyzed here as such, relying on the definition of public policy focused on political actors and their activities, notably that of Thomas Dye, for whom public policy is *“*whatever governments choose to do or not to do*”*[24]. The implementation of this policy was reportedly done by trial and error, with many inconsistencies. Human resource managers working in the districts and regions were facing difficulties in the sense that they had to respond to the concerns of the health officers, while they themselves did not have enough information about the policy. The absence of written directives appears to be one of the reasons for this “piecemeal” implementation. However, such a piecemeal policy implementation was not a unique situation. Ridde and Sardan also reported finding the same “chaotic” implementation of payment exemption policies in Burkina Faso, Mali, and Niger [31].

The development of this policy did not involve consensus building with all the health care system actors.

The policy was not based on scientific evidence, as the literature shows that the strategy of compulsory service is not efficient in the long term [4,6,7]. In fact, the regionalization of health personnel recruitment was akin to a regulatory policy with a compulsory contract. It forced regionalized workers to perform compulsory service in regions chosen, initially for an indeterminate period. In their classification of different public personnel retention policies, Wilson et al (2009) consider that coercive policies are weak and sometimes alienate health care professionals, who no longer wish to serve in remote regions in the long term [7].

While this type of policy is always time-limited for each officer and/or provides for motivational compensation [17,22], regionalization in Burkina Faso included no incentives. Regionalized workers received the same allowances as their non-regionalized colleagues. However, financial and non-financial compensation is necessary in a regulatory policy. Similar programs carried out elsewhere provide several kinds of financial incentives (e.g. hardship allowance, housing allowance, free transportation, paid leaves, etc.) as compensation for the drawbacks of job opportunities in remote areas [13,17,24]. Zambia implemented an incentive package to attract and retain doctors in rural and remote areas. The package included a rural location allowance of up to 30% of the basic salary, home renovation, contribution to children’s tuition fees, a vehicle, or support for rental housing, and future training [10].

Regionalized officers feel stigmatized in the sense that this policy does not apply to all health officers on contract with the State, but only to them, whereas both regionalized and non-regionalized nurses and midwives work for the Ministry of Health.

Like in Ecuador and in South Africa, health workers in our study complained about compulsory service, with regard to the management of their career [19,22]. Moreover, this policy often targeted recent graduates with little work experience [7,32].

Another limitation of the policy was that it does not extend to those qualified through the direct competitive process or to other categories of health professionals such as doctors and pharmacists. In fact, the demarcation between regionalized and non-regionalized workers hinges on how they gain access to the public service: direct selection based on a training school competitive process or selection after front-line worker training. The logic of this policy was not clear and as long as the issue of unequal distribution of health personnel in rural areas remains unsolved, other categories of workers should not be overlooked.

## Conclusions

After ten years of implementation, the health worker regional recruitment policy needs to be reviewed with the full participation of all actors, for improved implementation and efficacy. Its time limitation, the addition of financial and non-financial incentives, as well as its generalizability to all groups of health workers are conditions to be taken into account for its success.

## List of abbreviations used

CSPS: Centre de santé et de promotion sociale; DFATD: Foreign Affairs, Trade and Development Canada; GHRI: Global Health Research Initiative; IDRC: International Development Research Centre; MDG: Millennium Development Goal.

## Competing interests

The authors declare no competing interests.

## Authors’ contributions

VR and SK designed the study. SK wrote the introduction, the results, and the conclusion. WMEY wrote the methodology and the results. VR reviewed the manuscript. All authors performed reviews of drafts for intellectual content and approved the final manuscript.

## Declaration

The publication costs associated with this article are funded by Foreign Affairs, Trade and Development Canada and the International Development Research Centre through the Global Health Research Initiative.

This article has been published as part of *Human Resources for Health* Volume 12 Supplement 1, 2014: Addressing the human resources for health crisis through task-shifting and retention: results from the African Health Systems Initiative's research component. The full contents of the supplement are available online at http://www.human-resources-health.com/supplements/12/S1
